# Cause and Effect

**Published:** 1900-12-15

**Authors:** H. T. Harvey

**Affiliations:** Battle Creek, Mich.


### CAUSE AND EFFECT.

---

BY H. T. HARVEY, D.D.S., BATTLE CREEK, MICH.

---

Read at the Union Meeting of the Northern Indiana and Southwestern Michigan Dental Societies.

---

A discussion of the following subjects: 1. "Michigan Dental Statutes;" 2. "Michigan State Board of Examiners in Dentistry;" 3. "The Dental Profession;" 4. "The Illegitimate Practitioners of Dentistry."

I hesitated to prepare this paper or to speak before this assembly for two reasons: First, because my subject appeared to me to be more a matter of strictly Michigan interest; and, secondly, because I believed there were others who might better discuss the subject, and who had more time to devote to its preparation than myself. I refused when first invited, but out of respect to your Executive Committee and regard for the profession, I concluded my reasons for acceptance would be as follows: First, our Hoosier colleagues are entering Michigan more or less to practice dentistry, together with the fact that the report is becoming quite general that it is almost impossible legally to enter the State to practice dentistry, owing to the fact that such precautions have been taken that exclusion is the watchword of the day. Second, it has been reported quite generally that the writer has permanently retired from the practice of dentistry. I will disabuse the minds of all that neither is the case. I proclaim now to all that we cordially welcome all honorable dentists to join us, and I promise that if qualified to practice their chosen profession they will not be deterred, and will receive the hand of fellowship from all within our confines. I wish to state, here, that I am a dentist. I can never be anything else, I shall always be one of you, and continued devotion and allegiance to my profession is hereby pledged, prompted by a love for and a desire to elevate the same.

The Michigan Dental Statute was enacted in 1883 in this State. The primary cause for such enactment was the protection of the public. It was enacted by the public, not for any particular class, or by any particular class. The enactment of this statute provided a State Board of Examiners to pass upon the qualifications of those who desired to enter the State for practice, and did not possess a diploma from a reputable dental college. From 1883 to 1891 the diplomas of all regularly incorporated and reputable dental colleges (when I say reputable, I mean those belonging to the National Association of Dental Faculties), were registered by this State Board without examination. It afterwards became necessary to deny registration to graduates of certain colleges that used their franchises solely for pecuniary advancement, and to the almost utter demoralization of their students. For that reason there was enacted an amendment by our Legislature in 1891, *requiring* an examination of all persons who did not possess a diploma from a dental college whose curriculum was equal both in time and studies and the grade of examinations to the Dental Department of the University of Michigan. It was made the duty of the Board of Examiners to decide what colleges came under this classification. The Board, which was at that time composed of Drs. Metcalf, Corbin and Lathrop, decided that the following colleges had a course of instruction equal to the Dental Department of the University of Michigan, viz., the Dental Department of the University of California, Dental Department of Harvard and the Boston Dental College. Some three or four years later the Dental Department of the Detroit College of Medicine was accepted by the same Board.

As you well know, there has been a gross violation of this statute ever since its enactment. Numerous complaints have been made, both by members of the State Board and by individuals. Some of these cases have been cared for by the State Board, and others have been handled by local societies or by individuals. Until two or three years since, the majority of these prosecutions for some reason or other failed. Either the parties complained of evaded trial, or the case was poorly handled, and in some instances the prosecuting attorney did not co-operate with the complaining witness. These results had a demoralizing effect, and the dental profession always looked to the State Board for relief. The point that I wish to bring out in discussing our statute is first, although we assume that it is valid and that its validity will be sustained if passed upon by our Supreme Court, it never has come before that tribunal for its consideration and decision. I know that many of you will be surprised to learn this. The case of Allen vs. People was appealed from the Circuit Court to the Supreme Court last year, and a decision was rendered by the Supreme Court in December of the same year confirming the conviction of

Allen in the lower court, but in this decision the Supreme Court did not pass upon the statute but only on the technicality upon which the appeal was based. We have, however, received much benefit from the fact that this case before the Supreme Court was favorably decided.

I do not believe that failure to convict is likely to occur in a single case where the preliminary evidence is properly prepared and the prosecuting attorney of the county in which the complaint is made co-operates with the complaining witness. I also wish to express the belief that the validity of the statute, if passed upon by the Supreme Court of this State, will be upheld.

Why should the profession look to the State Board to take charge of cases of prosecution? As you well know, the Board was created by this same statute for the purpose of passing upon the qualifications of those who desire to register for the practice of dentistry. The position of all previous Boards has been that it is not the duty of the Board to make complaints against infraction of the dental statute, but on the contrary, it is the duty of members of the dental profession and other citizens. Why should this statute be enforced? Is it for the benefit of the dentists who are practicing in the State? Should not we really have a broader conception of dental legislation, and sincerely credit the public with the protection that it was originally intended to receive? Does the State Board benefit or do the individual members thereof benefit when there is a successful conviction of a violator of the statute? Do not the dentists who practice in the community in which the violation has occurred, benefit when such violation ceases?

Upon assuming the duties of a member of the State Board, I took this view of my official duties: first, that the State Board was created to represent the people of the State, and not the dental profession; secondly, that, although not expressed in legal terms, it should be the duty of the State Board to file all complaints in cases of prosecution against violators of the law, and to procure evidence sufficient to convict such violators. I also believe it to be the duty of the Board to request and accept the services of the prosecutor in the county in which the prosecution takes place. I further believe in admitting to practice in this State any person who can prove requisite qualifications to so practice, without regard to the school he may have attended, or whether he may have attended any, and without consulting the interests of any person or persons now engaged in the practice of dentistry in the State.

There has always been more or less friction between the Michigan State Dental Association and the State Board of Examiners in Dentistry. I have been a member of the Michigan Dental Association, which is supposed to represent the dental profession of the State, for a period of about nine years. I have never attended an annual session when the State Board was not only severely criticised, but many of their acts were seriously challenged. It appeals to my better judgment that if those dentists who aim to represent the profession of the State and who are combined together for such a purpose, would occasionally commend the members who serve gratuitously upon the State Board, for at least a part of their services there would be greater unanimity of feeling and a more cordial relation existing between the State Board and the dental societies. It is not pleasant after serving faithfully one's colleagues and the public at large for a period of three years upon such a board and having to pass the ordeals one is subjected to in the natural course of his duties, thinking to please his friends in the profession and serve them well, to learn that he is not even thanked. He has the gratitude of no one, and many times feels that he is even discredited. He is credited with all the ulterior motives that humanity is heir to, and criticised beyond reason. It has been a constant desire of the present State Board, to my positive knowledge, to co-operate in detail and consult with the dental profession of this State. I do not presume that you members of the profession of the State who are present here to-day appreciate quite as thoroughly as I can the continued onslaught of rebuffs that have constantly been poured in upon the Board for the past year and a half.

There have been some eight or ten arrests made within this period of time for the violation of the statute and in every instance the State Board has had charge of the case, and in each case the person complained of has either retired from the State, ceased to practice or has been convicted in the courts.

The Board discovered early last year that not only were the unqualified violating the law, but many whose privilege it was to register by payment of a fee of $3 and filing an affidavit with the Board, according to law, were not only neglecting to so register, but even defied the Board. The only cases that came to the notice of the Board were those who were graduates of the two schools in the State. In order to test that phase of the statute, I complained of a graduate of the University of Michigan who had repeatedly refused to comply with the law and who defied the authority of the Board, with the result that he was convicted in the Police Court in the city of Detroit, last December. Almost immediately there was a great influx of graduates from both of these colleges to register. Indeed, to-day, I do not believe that there are many graduates who locate within the State who do not register before locating and avail themselves of the first opportunity to do so.

I wish in this connection to make the assertion, that

Michigan is more free from illegitimate practitioners than it has been since the enactment of our first law in 1883. To my knowledge there are some dozens of dentists who were located in this State a year and a half ago, who have retired to other States to practice. I wish also to make the assertion that there are fewer graduates of colleges whose diplomas are registerable by the Board practicing in the State without registration than there has been since 1891; credit for this improvement should be given to the Board. I do not pretend to say that the State is entirely free from illegal practitioners, but I mean to say that if there is a dental organization in this State, or an individual who really will make a business of promoting the welfare of the profession as such, there need not be a violator of the law practicing in the State. And any individual or organization may co-operate with the Michigan State Board of Dental Examiners as it is now constituted assured of having the personal and official co-operation in suppressing illegitimate practitioners. I may further add that there has been an absolute unanimity in all decisions and in all acts by the State Board since its present organization.

I want to refer briefly to violators of our dental law as we find them. They enter the State generally with a knowledge that they must violate our dental statute. There are cases where they really do not know this until after they become located. It is usually that class of people which is unsuccessful, as a rule, in other localities, men who have used all their money to get their equipment and in consequence, when they have become located, are without funds to remove to other locations or take up new avenues of business. By the time the fact becomes known that they are practicing in the community without a license, they have formed a circle of friends. In most instances these friends become more constant when they find that the violator is in trouble. It seems to be a long process before the State Board is advised of these infractions. By the time sufficient evidence is procured upon which to base a warrant for arrest the infractor has a very large circle of sympathizers, and without exception if the case is brought to trial in the community in which he has been practicing, some of these sympathizers are likely to be upon the jury. In many instances these violators have families, the wives and children of which are respected and for whom there is a general expression of pity. There is always more or less influence brought to bear upon the prosecuting attorney whose duty it is to take charge of these prosecutions, and in many instances it consumes a large amount of time and forcible arguments to induce the prosecuting attorney to have anything to do with the case at all. In fact, I have come across two prosecuting attorneys who absolutely refused to prosecute, and it was only upon the threat that they would be complained of to the Governor by the Board if they continued to refuse to perform the duties of their office that they were induced to take up the cases. I assure you it is not a pleasant task to become in the eyes of the public persecutors, whose only vocation it appears to be to dog the more unfortunate of our own class. I am simply closing this paper with a few of the unpleasant features that a member of this Board meets in the discharge of his duties. You have had the rosy side of it in times past in listening to discussions of how easy it was for a politician to become a member of this Board and the honors and the large amount of money he received for his services after becoming a member of the Board. Still the pleasure one receives in knowing that he has faithfully discharged his duties to the best of his ability is sufficient to repay for all the unpleasantnesses that may arise from time to time. I do not wish to imply that this Board is above criticism, we solicit most cordially any just criticism. We are not infallible. We do not pretend to be. We are men, and like other men we make mistakes. We are willing to have these errors pointed out to us.

Again I wish to remind you that there is an opportunity for a more harmonious state of affairs in our midst, and I trust that any obstacles that are now in the way may be removed, to the end that we, as a profession, may become united, and may move forward in one solid body. When this quibbling ceases we will respect ourselves more, and we will have the respect of our fellowmen to a greater degree than now. As a unit, the force and influence of which will be felt, we may be a power for good. Do not forget that Michigan is looked to in various channels of our profession as a criterion. Michigan is often referred to in the National Board of Examiners' meetings and also in the National Association of Dental Faculties, and also in matters of dental legislation, both local and national.

